# Paternal Preconception Chronic Variable Stress Confers Attenuated Ethanol Drinking Behavior Selectively to Male Offspring in a Pre-Stress Environment Dependent Manner

**DOI:** 10.3389/fnbeh.2018.00257

**Published:** 2018-11-02

**Authors:** Gregory R. Rompala, Alison Simons, Brooke Kihle, Gregg E. Homanics

**Affiliations:** ^1^Center for Neuroscience, University of Pittsburgh School of Medicine, Pittsburgh, PA, United States; ^2^Department of Neuroscience, University of Pittsburgh, Pittsburgh, PA, United States; ^3^Department of Pharmacology and Chemical Biology, University of Pittsburgh School of Medicine, Pittsburgh, PA, United States; ^4^Department of Anesthesiology, University of Pittsburgh School Medicine, Pittsburgh, PA, United States; ^5^Department of Neurobiology, University of Pittsburgh School of Medicine, Pittsburgh, PA, United States

**Keywords:** stress, ethanol, intergenerational, ethanol drinking, epigenetics

## Abstract

Stress-related psychiatric disorders such as major depression are strongly associated with alcohol abuse and alcohol use disorder. Recently, many epidemiological and preclinical studies suggest that chronic stress prior to conception has cross-generational effects on the behavior and physiological response to stress in subsequent generations. Thus, we hypothesized that chronic stress may also affect ethanol drinking behaviors in the next generation. In the first cohort of mice, we found that paternal preconception chronic variable stress significantly reduced both two-bottle choice and binge-like ethanol drinking selectively in male offspring. However, these results were not replicated in a second cohort that were tested under experimental conditions that were nearly identical, except for one notable difference. Cohort 1 offspring were derived from in-house C57BL/6J sires that were born in the animal vivarium at the University of Pittsburgh whereas cohort 2 offspring were derived from C57BL/6J sires shipped directly from the vendor. Therefore, a third cohort that included both in-house and vendor born sires was analyzed. Consistent with the first two cohorts, we observed a significant interaction between chronic stress and sire-source with only stressed sires that were born in-house able to impart reduced ethanol drinking behaviors to male offspring. Overall, these results demonstrate that paternal preconception stress can impact ethanol drinking behavior in males of the next generation. These studies provide additional support for a recently recognized role of the paternal preconception environment in shaping ethanol drinking behavior.

## Introduction

Over the last several years, there has been a burgeoning interest in epigenetic inheritance; that is, the transfer of acquired traits from parent to child via nongenomic germline mechanisms. For instance, several epidemiological findings suggest that preconception stress affects offspring development and stress sensitivity (Bowers and Yehuda, 2016). This hypothesis is supported by wide-ranging preclinical studies illustrating that the paternal preconception environment imparts diverse behavioral phenotypes to offspring as recently reviewed in Chan et al. (2018). As these studies are largely carried out with isogenic mice or rats, the intergenerational effects are unlikely to be explained by genetic variation. Therefore, additional studies are needed to elucidate novel epigenetic mechanisms of inheritance with major human health implications.

Several animal models of early life and chronic stress including maternal separation (Franklin et al., 2010), social defeat (Dietz et al., 2011), social isolation (Pisu et al., 2013) and chronic variable stress (Rodgers et al., 2013) have been found to directly influence complex neurobehavioral phenotypes in offspring. In addition, paternal chronic corticosterone (CORT) exposure increases anxiety-related behaviors in offspring (Short et al., 2016) and many of these paternal stress exposures alter hypothalamic-pituitary-adrenal (HPA) axis function in offspring (Dietz et al., 2011; Pisu et al., 2013; Rodgers et al., 2013; Gapp et al., 2016).

As with paternal preconception stress, there have been several paternal preconception ethanol exposure studies in rodents which have found a wide range of physiological and behavioral effects in offspring (for review see Finegersh et al., 2015). Recently, we discovered that paternal chronic ethanol vapor exposure blunted HPA axis responsivity in male offspring (Rompala et al., 2016), similar to the cross-generational effects of chronic stress (Rodgers et al., 2013). Given that ethanol directly engages the HPA axis (Rivier, 2014), it is conceivable that ethanol and stress act through convergent mechanisms to impart intergenerational phenotypes.

Notably, the same paternal ethanol exposure that blunts stress responsivity (Rompala et al., 2016), also confers reduced ethanol drinking and increased ethanol behavioral sensitivity selectively to male offspring (Finegersh and Homanics, 2014; Rompala et al., 2017). Thus, if the HPA axis-engaging mechanisms of ethanol underlie the effects of paternal ethanol exposure, other forms of chronic stress may similarly be able to influence intergenerational ethanol drinking behaviors. Indeed, chronic stress promotes ethanol drinking behavior (Becker et al., 2011), but the cross-generational relationship between paternal chronic stress and ethanol drinking behavior remains unexplored.

Given the high prevalence and societal costs associated with alcohol use disorder (Rehm et al., 2009; Haberstick et al., 2014), the potential for paternal preconception environment to causally affect intergenerational ethanol-related behaviors warrants further investigation. Thus, the current study examined the hypothesis that paternal chronic variable stress impacts the ethanol drinking phenotype of the next generation. Here, we discover a novel effect of paternal stress on offspring ethanol drinking behavior and underscore the crucial role of sire pre-stress environment in the manifestation of intergenerational phenotypes.

## Materials and Methods

All experiments were approved by the Institutional Animal Care and Use Committee of the University of Pittsburgh and were conducted in accordance with the National Institutes of Health Guidelines for the Care and Use of Laboratory Animals.

### Animals

Seven-week-old, C57BL/6J (B6) and Strain 129S1/SvImJ (Strain 129) mice were purchased from the Jackson Laboratory (Bar Harbor, ME, USA). Animals at Jackson Laboratory were weaned into group-housing by sex at 10–20 animals/cage. Animals from different litters were mixed together at weaning such that cage-mates include siblings and non-siblings. Upon arrival at the University of Pittsburgh, unless otherwise specified, specific pathogen-free mice were group-housed to 3–4 mice/cage in individually ventilated micro-isolater cages under 12-h light/dark cycles (lights on at 07:00) and had *ad libitum* access to food (irradiated 5P76 ProLab IsoPro RMH 3000 (LabDiet, St. Louis, MO, USA)) and water.

### Establishing Sire Cohorts for Paternal Chronic Variable Stress

*For generation of in-house sire colony*: B6 male mice purchased from Jackson Laboratory were habituated 6 weeks before breeding for 1 week to purchased 8-week-old B6 females (habituated 1 week) to produce the in-house colony cohort from the University of Pittsburgh (PITT). *For rearing and weaning of eventual in-house-sires*: after the 1 week breeding period, male breeders were removed and each pregnant female was moved to individual housing. At 3 weeks post-natal, eventual in-house-sires were weaned to four males per cage before the onset of chronic stress at 8 weeks postnatal. *For vendor-born and -shipped (JAX)-sires*: B6 male mice purchased at 7 weeks of age were habituated for 1 week to the animal vivarium before chronic stress or control treatment.

### Paternal Preconception Chronic Variable Stress

Eight-week-old adult male B6 group-housed mice were exposed to 6 weeks of chronic variable stress or control conditions. The chronic variable stress exposure was based on published methods (Rodgers et al., 2013, 2015). Briefly, chronic variable stress consisted of daily exposure to one of seven stressors (each described below) on a randomized schedule with each stressor utilized at least once per 7 days and six times in total. For both control and stress exposure groups, body weights were measured and cages were changed simultaneously and weekly at the same time of day (09:00–11:00).

### Novel Object Exposure

Thirty glass marbles (10 mm diameter) were placed in the home cage for 12 h during the dark cycle.

### Saturated Cage Bedding Overnight

At the onset of the dark cycle, ~200–600 ml (depending on the amount of bedding in the cage) of autoclaved water (~23°C) was applied to the home cage. The exposure was terminated at the onset of the light cycle when the home cage was changed and mice were gently dried with a towel and then briefly (~5 min) the open cage was placed under a heat lamp.

### White Noise Overnight

From the onset to the termination of the dark cycle (12 h ± 30 min), home cages were moved to sound-controlled chambers in an animal behavior room (same room each exposure) fitted with a ventilation fan and computer-operated speakers programmed with Audacity 2.2.1 software to emit continuous 100 db white noise.

### Multiple Cage Changes

Throughout the 12 h light cycle, home cages were changed 3–5 times at randomized time points.

### Constant Light Exposure

Home cages were placed in an air-controlled and ventilated fan-equipped clear plexiglass chamber with room lights left on from the onset to the termination of one dark cycle.

### Restraint Stress

Animals were restrained for 15 min between 3 h and 5 h after lights-on (10:00–12:00). Mice were restrained in 50 ml conical plastic tubes (VMR, Radnor, PA, USA Cat# 525–0158) with several air hole perforations near the animal’s head. All mice in the group-housed cage were restrained within the home cage simultaneously in a fume hood.

### Predator Odor Exposure

The predator odor exposure was performed in the home cage within a fume hood with the cage cover removed. All stress-treatment cages were exposed simultaneously for 15 min with 3 × 3 inch article towel strips soaked with 1 mL of fox urine (Tink’s Red Fox-P^®^, Tink’s, Covington, GA, USA; Cat# W6245) placed just outside each cage, flanking each length-wise side of the cage.

### Breeding Scheme and Offspring Rearing

Following the final chronic variable stress exposure, mice were pair housed with purchased 8-week-old B6 females for 2 weeks. This 2-week period was chosen to: (a) control for any effects of acute stress prior to breeding on maternal care for the offspring; and (b) allow eventual sires to purge older sperm that matured prior to the onset of chronic stress exposure. Plasma CORT levels were measured 1 week following the final stressor and 2 h before the start of the dark cycle (17:00) using the CORT ELISA kit (Cat# ADI-900-097; Enzo Life Sciences, Farmingdale, NY, USA). After the 2-week post-stress period, all males were moved to housing with two stress-naïve Strain 129 8-week-old females for 48 h before males were removed and pregnant dams were single-housed for rearing of stress (S) and control (C)-sired offspring. Offspring were weaned at 3 weeks postnatal and group-housed (3–4/cage) with same sex littermates of the same treatment group. Importantly, for all behavioral testing, at least one and no more than two mice of the same sex were examined per litter and per sire.

### Twenty-Four Hour, Two-Bottle Free Choice Drinking Behavior

Eight-week-old adult mice were single-housed for 1 week while habituating to two 25 ml sipper tubes filled with autoclaved water. Sipper tubes were designed by fitting ball-bearing sippers (Cat# TD-99; Ancare Corp, Bellmore, NY, USA) into modified 25 ml polystyrene serological pipets (Cat# 357525; Corning Incorporated, Corning, NY, USA) and securing the fit with heat-shrink and parafilm. After the 1 week habituation, ethanol drinking behavior was assessed by filling one tube with ethanol. Consumption of ethanol and water was measured daily, and the position of the ethanol and water tubes was rotated each day. Ethanol concentrations started at 3% (w/vol) and was increased every 4 days to 6, 9, 12 and 15% successively. Cages were changed and animals were weighted every 4 days when the ethanol concentrations were adjusted. After the final day of ethanol drinking, there was a 1-week washout period, during which mice had access to two sipper tubes filled with water. After the washout period, one tube was filled with 0.06% (g/ml) saccharin (Cat# 240931; Sigma-Aldrich, St. Louis, MO, USA) and two bottle consumption was measured on each of the four trial days. After the final trial, there was another 1-week washout before one tube was filled with 0.06 mM quinine (Cat# 145904; Sigma-Aldrich, St. Louis, MO, USA) and two bottle consumption was measured on each of the four trial days. Tube positions were rotated daily.

### Drinking in the Dark Assay

The drinking in the dark assay was performed based on published methods (Thiele et al., 2014). For four nights, single-housed mice were habituated in the home cage to one 25 ml sipper tube filled with water that replaced their regular water bottle 2 h into the animal’s dark cycle. After the final habituation trial, sipper tubes were filled with 20% (w/vol) ethanol and consumption was measured for 2 h training trials over three consecutive days and for 4 h in a test trial on the final day.

To examine blood ethanol concentrations (BEC) immediately following the test trial, ≤10 μL tail vein blood was collected from each animal using heparin-coated capillary tubes (Cat# 1-000-3200-H; Drummond, Broomall, PA, USA). Tail blood was centrifuged at 2,000× *g* for 10 min and plasma was stored at −80°C prior to being measured for ethanol (mg/dL) using an AM1 Analox Ethanol analyzer (Analox Instruments, London, UK).

### Hypothalamic-Pituitary-Adrenal (HPA) Axis Responsivity

During the animal’s light cycle between 10:00 and 13:00, single-housed mice were restrained for 15 min in modified 50 ml conical tubes with the cone endings removed and an aperture added to the cap for the tail. Tail blood was collected with heparin-coated capillary tubes (Drummond) at time points 0, 15, 30 and 90 min from the onset of restraint stress. Plasma was collected from blood by centrifugation at 2,000× *g* for 10 min. CORT levels were measured in plasma using CORT ELISA kit (Enzo Life Sciences). Using 5 μL of plasma, samples were prepared and analyzed in duplicate on a 96-well plate following manufacturer’s protocol.

### Statistical Analysis

Saccharin and quinine drinking preference were analyzed using two-way unpaired Student’s *t*-test. Because body weights and ethanol drinking behaviors are known to differ between sexes, male and female (Tambour et al., 2008) results were analyzed separately. Body weights, two-bottle choice ethanol drinking, drinking in the dark, and BEC results were analyzed with two or three-way (i.e., paternal stress × sire source × ethanol concentration or trial number) repeated measures analysis of variance (ANOVA). Drinking in the dark results were analyzed with two-way repeated measures ANOVA by averaging the three 2h trials to produce two conditions for trial length (2 and 4 h). Litter size results were analyzed using Student’s *t-tests* or two-way ANOVA (factors of paternal stress and sire source). Significant interactions were further analyzed using Fisher’s least significant difference (LSD) *post hoc* tests. Basal CORT levels were compared using two-way ANOVA (factors of paternal stress and sire source) and HPA axis responsivity was assessed using repeated measures two-way ANOVA (factors of paternal stress and time point). All data are presented as mean ± standard error of the mean. All analyses were performed with GraphPad Prism 6 (GraphPad Software, La Jolla, CA, USA) or Statistica v10 (TIBCO Statistica, Palo Alto, CA, USA) software.

## Results

### Paternal Preconception Chronic Variable Stress

**Figure 1:**
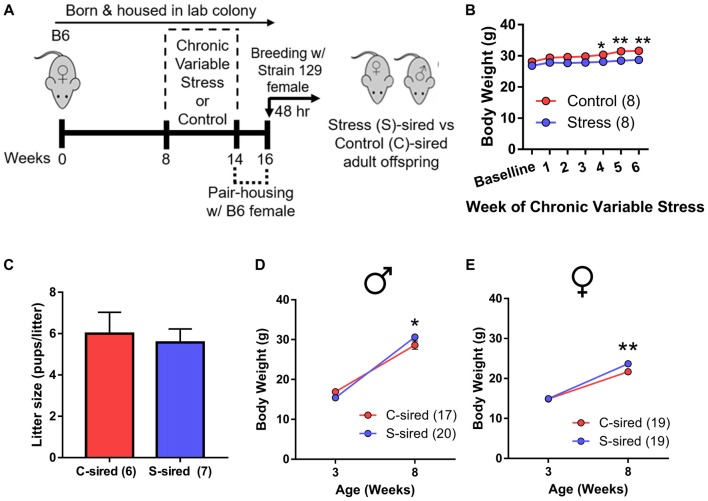
Intergenerational effects of paternal preconception chronic stress. **(A)** Experimental timeline. Eight-week-old male mice were exposed to 6 weeks of chronic variable stress or control conditions. Two weeks after chronic variable stress or control conditions, males were bred with stress-naïve Strain 129 females for two nights to produce male and female stress (S)-sired and control (C)-sired offspring. **(B)** Significantly reduced body weights in S-sires vs. C-sires (*N* = 8/group). **(C)** No effect of paternal stress on litter sizes (*N* = 6–7/group). **(D)** Increased body weight at 8 weeks postnatal in S-sired vs. C-sired male offspring (*N* = 17–20/group). **(E)** Increased body weight at 8 weeks postnatal in S-sired vs. C-sired female offspring (*N* = 19/group). *p < 0.05, **p < 0.01. Error bars in panels **(B,D,E)** are obscured by symbols.

Adult B6 male mice were either exposed to 6 weeks (1 week longer than the spermatogenic cycle of mice (Jones, 1999) of chronic variable stress or control conditions. To control for the acute effects of stress and to purge older sperm that may have matured prior to the chronic stress exposure (Rodgers et al., 2013), males were caged with one B6 female for 2 weeks immediately following chronic stress or control treatment. Immediately following this 2 week mating period, each male was caged for two nights with two stress-naïve Strain 129 breeder females to produce stress (S)-sired and control (C)-sired male and female offspring (Figure 1A). Strain 129 females were utilized to remain consistent with previous studies examining intergenerational effects of stress and ethanol exposures (Rodgers et al., 2013; Finegersh and Homanics, 2014; Rompala et al., 2016). See Supplementary Table S1 for a complete summary of all experimental cohorts in the current study.

Analysis of sire body weight prior to mating revealed significant main effects of week of exposure (*F*_(6,42)_ = 4.63, *p* < 0.01) and chronic stress on body weight (*F*_(1,7)_ = 63.16, *p* < 0.001; Figure 1B), but no week of exposure × chronic stress interaction. *Post hoc* analysis revealed that chronic stress reduced body weights at weeks 4 (*p* < 0.05), 5 (*p* < 0.01) and 6 (*p* < 0.01) of exposure. There was no effect of paternal stress on litter size (Figure 1C). There was a significant effect of postnatal age on body weights for both male (*F*_(1,35)_ = 476.30, *p* < 0.001) and female (*F*_(1,36)_ = 302.90, *p* < 0.001) offspring with no main effect of paternal stress. However, there was a significant paternal stress × postnatal age interaction for both male (*F*_(1,35)_ = 8.12, *p* < 0.01, Figure 1D) and female (*F*_(1,36)_ = 4.62, *p* < 0.05; Figure 1E) offspring body weight. *Post hoc* analysis revealed that stress did not affect body weigh at 3 weeks postnatal, but increased S-sired male (*p* < 0.05) and female (*p* < 0.01) weights at 8 weeks postnatal.

### Paternal Stress Reduces Ethanol Drinking Preference in Males

**Figure 2:**
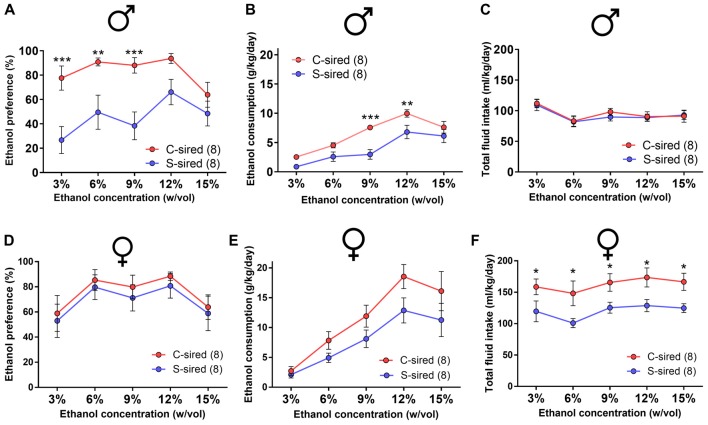
Paternal stress reduces ethanol drinking behavior in males. For male offspring (*N* = 8/group), paternal stress significantly reduced **(A)** ethanol preference and **(B)** ethanol consumption and had no effect on **(C)** total fluid intake. For female mice (*N* = 8/group), there was no effect of paternal stress on **(D)** ethanol preference or **(E)** ethanol consumption, and a significant reduction in **(F)** total fluid intake. *p < 0.05, **p < 0.01, ***p < 0.001. Each data point represents the daily average calculated from four 24h trials.

Adult S-sired and C-sired animals were examined for ethanol drinking preference in a two-bottle free choice test at sequential ethanol concentration of 3, 6, 9, 12 and 15% (w/vol) for 4 days at each concentration. In males, there was a significant main effect of paternal stress on ethanol drinking preference (*F*_(1,35)_ = 37, *p* < 0.001; Figure 2A). *Post hoc* tests revealed that paternal stress reduced ethanol preference at 3% (*p* < 0.001), 6% (*p* < 0.01), and 9% (*p* < 0.001) ethanol concentrations. There was a significant effect of paternal stress on ethanol consumption (*F*_(1,14)_ = 11.24, *p* < 0.01, Figure 2B) and *post hoc* tests revealed significant reductions at 9% (*p* < 0.001) and 12% (*p* < 0.01) ethanol concentrations. There was no effect or paternal stress on total fluid intake (*p* > 0.05, Figure 2C). There was a significant effect of ethanol concentration on ethanol consumption (*F*_(4,56)_ = 38.77, *p* < 0.001) and total fluid intake (*F*_(4,56)_ = 12.72, *p* < 0.001). There was no interaction between paternal stress × ethanol concentration for ethanol preference, ethanol consumption, or total fluid intake. For female offspring, there was no effect of paternal stress on ethanol preference (*p* > 0.05, Figure 2D) or ethanol consumption (*p* > 0.05; Figure 2E), but there was a significant effect of paternal stress on total fluid intake (*F*_(1,14)_ = 8.59, *p* < 0.05; Figure 2F). *Post hoc* tests revealed that paternal stress significantly reduced total fluid intake at all ethanol concentrations in S-sired females (*p* < 0.05). There was a significant effect of ethanol concentration on ethanol preference (*F*_(4,56)_ = 5.81, *p* < 0.001), ethanol consumption (*F*_(4,56)_ = 34.59, *p* < 0.001) and total fluid intake (*F*_(4,56)_ = 2.60, *p* < 0.05). There was no significant effect of paternal stress × ethanol concentration on ethanol preference, ethanol consumption, and total fluid intake. There was no effect of paternal stress on male or female drinking preference for saccharin or quinine tastants (*p* > 0.05; Supplementary Figures 1A,B).

### Paternal Stress Reduces Binge-Like Ethanol Drinking in Males

**Figure 3:**
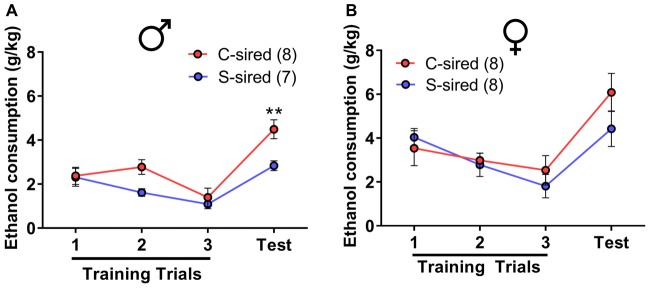
Paternal stress reduces binge-like ethanol consumption in males. **(A)** Reduced ethanol consumption in the drinking in the dark assay in S-sired males compared to C-sired males (*N* = 8/group). **(B)** No effect of paternal stress on ethanol consumption in females (*N* = 8/group). **p < 0.01.

The effects of paternal stress on ethanol drinking behavior were further assessed using the drinking in the dark model for “binge-like” ethanol consumption (Thiele et al., 2014). In this assay, animals have access to 20% ethanol in 2 h training trials conducted over three consecutive days followed by a final 4 h test trial day. There was a significant effect of trial on ethanol consumption in male (*F*_(3,39)_ = 17.15, *p* < 0.001) and female offspring (*F*_(3,42)_ = 14.18, *p* < 0.001). In male offspring, there was a significant attenuating effect of paternal stress on ethanol consumption (*F*_(1,13)_ = 13.12, *p* < 0.01; Figure 3A). *Post hoc* tests revealed that chronic stress reduced ethanol consumption during the 4 h test trial (*p* < 0.01). In female offspring, there was no effect of paternal stress on ethanol consumption (*p* > 0.05; Figure 3B). There was no paternal stress × trial interaction for either sex.

### No Effect of Paternal Stress on HPA Axis Responsivity

**Figure 4:**
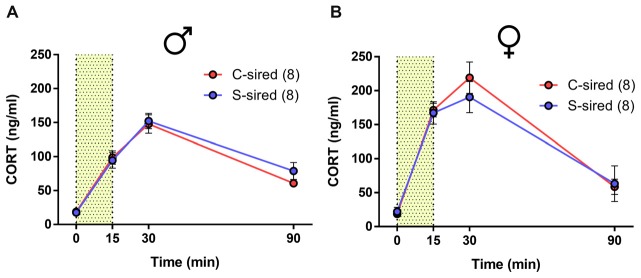
No effect of paternal chronic stress on hypothalamic-pituitary-adrenal (HPA) responsivity in offspring. No effect of paternal stress on **(A)** male (*N* = 8/group) or **(B)** female (*N* = 8/group) corticosterone (CORT) levels at 0, 15 30 and 90 min from the onset of 15-min restraint stress (represented by shaded bar).

Previous studies found that paternal preconception chronic variable stress suppressed acute restraint stress-induced CORT levels in male and female offspring (Rodgers et al., 2013, 2015). Similarly, we previously reported that paternal preconception chronic ethanol exposure reduced HPA axis responsivity, but only in male offspring (Rompala et al., 2016). In the present study, there was a significant effect of time from onset of 15 min of acute restraint stress on CORT levels for males (Figure 4A; *F*_(3,42)_ = 88.18, *p* < 0.001) and females (Figure 4B; *F*_(3,36)_ = 55.73, *p* < 0.001), but there was no effect of paternal stress or paternal stress × time interaction for either sex.

### No Effects of Paternal Stress in Offspring of Sires Procured From Vendor

**Figure 5:**
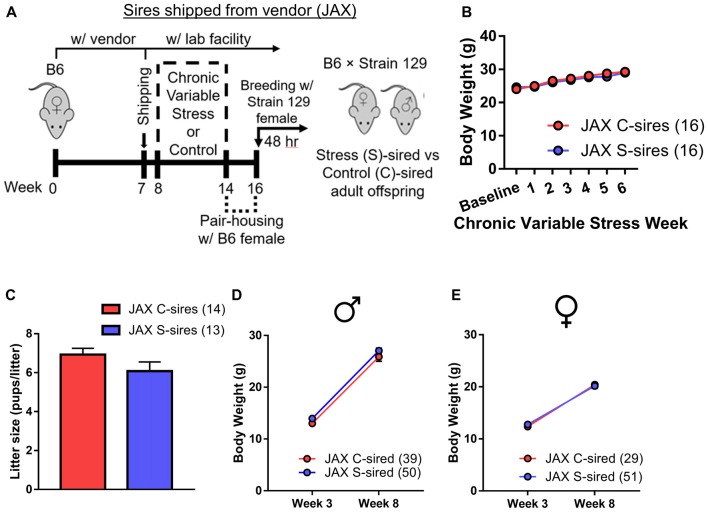
Paternal chronic stress with vendor (JAX)-shipped sires. **(A)** Sires were shipped from JAX at 7 weeks postnatal to the animal vivarium before being exposed to chronic stress from 8 weeks to 14 weeks postnatal and, at 16 weeks, were bred to Strain 129 females to produce JAX S-sired and C-sired male and female offspring. **(B)** No effect of chronic stress on sire body weight (*N* = 16/group). **(C)** No effect of paternal stress on litter sizes (*N* = 13–14/group). **(D)** No effect of paternal stress on body weights of JAX-sired males (*N* = 39–50/group) or **(E)** JAX-sired females (*N* = 29–51/group). Error bars are obscured in panels **(B,D,E)**.

A second study was conducted on an independent cohort of animals in an attempt to replicate the results reported above. All experimental conditions were identical to the first cohort study, except for one seemingly unimportant difference. The first cohort of paternal stress sires was the second generation of an in-house colony, housed within the animal vivarium at the University of Pittsburgh School of Medicine from birth (see Figure 1C). In the follow-up second cohort, sires were born with the vendor (The Jackson Laboratory (JAX), Bar Harbor, ME, USA) and shipped to the animal vivarium 1 week prior to the onset of chronic stress or control conditions (Figure 5A).

In this second cohort of JAX-born sires, we observed a significant effect of week of exposure on body weight (*F*_(6,206)_ = 46.38, *p* < 0.001), but no effect of stress and no stress × week interaction for body weight (Figure 5B). There was no effect of paternal stress on litter size (Figure 5C). For offspring from JAX sires, there was an effect of postnatal age on body weight for male (Figure 5D; *F*_(1,104)_ = 556, *p* < 0.001) and female (Figure 5E; *F*_(1,96)_ = 427.3, *p* < 0.001) offspring, but no effect of paternal stress or paternal stress × postnatal age interaction for male or female offspring body weight.

**Figure 6:**
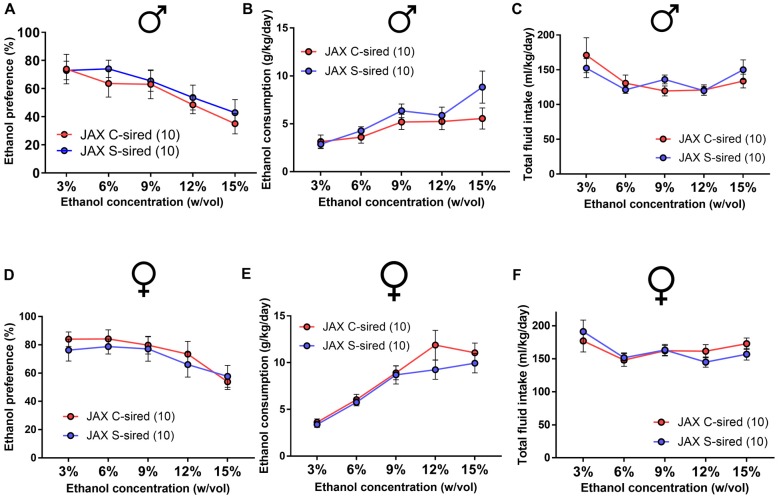
No effect of JAX-sire paternal stress on offspring ethanol drinking. No effect of paternal stress on ethanol preference, ethanol consumption and total fluid intake of **(A–C)** males (*N* = 10/group) or **(D–F)** females (*N* = 10/group) in the two-bottle, free choice drinking assay.

As in the initial cohort, the effect of paternal stress on two-bottle free choice ethanol drinking behavior in JAX-sired offspring was examined. There was a significant main effect of ethanol concentration for both sexes on ethanol preference (males: *F*_(4,72)_ = 8.86, *p* < 0.001; females: *F*_(4,72)_ = 9.46, *p* < 0.001), ethanol consumption (males: *F*_(4,72)_ = 8.15, *p* < 0.001; females: *F*_(4,72)_ = 38.65, *p* < 0.001) and total fluid intake (males: *F*_(4,72)_ = 4.43, *p* < 0.01; females: *F*_(4,72)_ = 6.07, *p* < 0.001). However, there was no effect of paternal stress and no paternal stress × ethanol concentration interaction for male (Figures 6A–C) or female (Figures 6D–F) ethanol preference, ethanol consumption, or total fluid intake.

**Figure 7:**
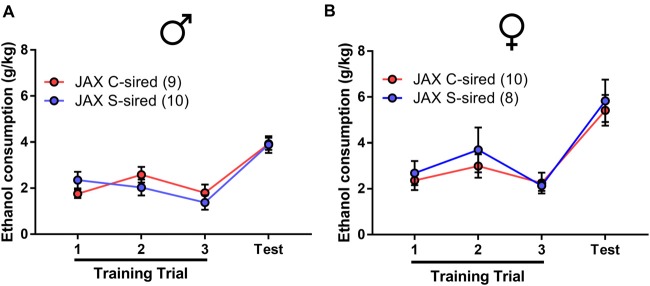
No effect of JAX sire paternal stress on binge-like ethanol drinking. No effect of paternal stress with JAX sires on ethanol consumption of **(A)** males (*N* = 9–10/group) or **(B)** females (*N* = 10/group) in the drinking in the dark assay.

In the drinking in the dark assay (Figure 7), there was a significant effect of trial on male (*F*_(3,48)_ = 17.68, *p* < 0.001) or female offspring (*F*_(3,48)_ = 17.68, *p* < 0.001). However, there was no effect of paternal stress and no paternal stress × trial interaction for ethanol consumption in either sex.

### Intergenerational Effects of Paternal Stress Are Dependent on Source of Sires

As reported above, we observed cohort-dependent effects of paternal preconception stress on ethanol preference and consumption. The experimental conditions between cohorts were nearly identical except for one notable exception: whereas cohort 1 offspring were derived from in-house sires born in the animal vivarium at the University of Pittsburgh (PITT), cohort 2 offspring were derived from JAX-shipped sires. Therefore, a third cohort that included both PITT and JAX sires was used to directly test the hypothesis that the effect of paternal preconception stress on offspring ethanol drinking were dependent on sire source. Only adult male offspring were studied due to cohort size limitations and the absence of ethanol phenotypes in females in either of the first two cohorts.

**Figure 8:**
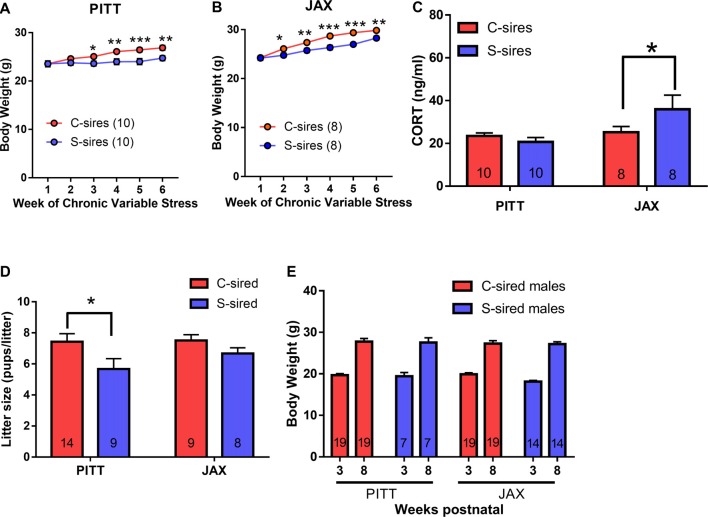
Comparing the effects of stress on in-house colony (PITT) vs. JAX-shipped sires. **(A)** Stress significantly reduced sire body weights in PITT and **(B)** JAX sires (*N* = 8–10/group). **(C)** Significant increase in basal CORT levels in JAX S-sires compared C-sires (*N* = 8–10/group). **(D)** Significant effect of paternal stress on litter sizes (*N* = 8–14/group). **(E)** No effect of paternal stress on body weights at three and 8 weeks postnatal (*N* = 7–19/group). *p < 0.05, **p < 0.01, ***p < 0.001. Error bars are obscured by data points in panels **(A,B)**.

For sire body weights, there was a significant main effect of sire source (*F*_(1,5)_ = 24.33, *p* < 0.001), there was no sire source × stress interaction and there were no week of exposure interactions with paternal stress or sire source. Therefore, PITT and JAX cohorts were examined separately. For both PITT (Figure 8A) and JAX sires (Figure 8B), there were significant effects of week of exposure (PITT: *F*_(5,90)_ = 15.35, *p* < 0.001; JAX: *F*_(5,70)_ = 51.73, *p* < 0.001), chronic stress (PITT: *F*_(1,18)_ = 6.9, *p* < 0.05; JAX: *F*_(1,14)_ = 54.07, *p* < 0.001), and week of exposure × chronic stress (PITT: *F*_(5,90)_ = 5.01, *p* < 0.001; JAX: *F*_(5,70)_ = 2.86, *p* < 0.05) on body weights. *Post hoc* analysis revealed significantly reduced body weights at weeks 3 (*p* < 0.05), 4 (*p* < 0.01), 5 (*p* < 0.001) and 6 (*p* < 0.01) for PITT S-sires vs. C-sires and reduced body weights at weeks 2 (*p* < 0.05), 3 (*p* < 0.01), 4 (*p* < 0.001) 5 (*p* < 0.001) and 6 (*p* < 0.01) for JAX S-sires vs. C-sires.

To examine the effect of chronic stress on basal CORT levels (i.e., in the absence of an acute stressor) in sires 1 week after the final chronic variable stress exposure, there was no effect of chronic stress, but a significant effect of sire source (*F*_(1,32)_ = 6.0, *p* < 0.05; Figure 8C) and a trending effect of chronic stress × sire source (*F*_(1,32)_ = 3.9, *p* < 0.06). *Post hoc* analysis of the chronic stress × sire source interaction trend (*p* < 0.06) revealed that chronic variable stress significantly increased plasma CORT levels in JAX S-sires vs. JAX C-sires (*p* < 0.05), but not PITT S-sires vs. PITT C-sires.

There was a significant main effect of paternal stress on litter size (*F*_(1,36)_ = 5.83, *p* < 0.05; Figure 8D) and no effect of sire source and no paternal stress × sire source interaction. *Post hoc* test revealed significantly reduced litter size in PITT S-sired vs. C-sired offspring (*p* < 0.05). For offspring body weights, there was no effect of paternal stress, sire source, paternal stress × sire source interaction and no postnatal week interaction with paternal stress or sire source (Figure 8E).

### Paternal Chronic Stress Reduces Two Bottle, Unlimited Access Ethanol Consumption Selectively in PITT-Sired Offspring

**Figure 9:**
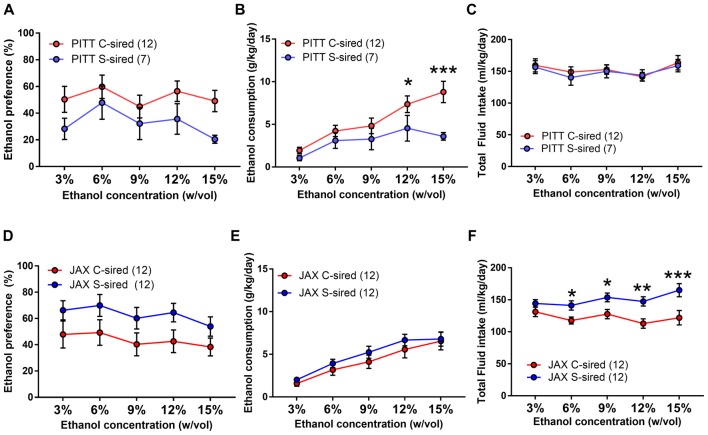
Paternal stress reduces ethanol drinking selectively in PITT S-sired offspring. **(A)** No change in ethanol preference of PITT S-sired male offspring. **(B)** Significant reduction in ethanol consumption in PITT S-sired male offspring. **(C)** No effect of paternal stress on total fluid intake in PITT sired male offspring. No effect of paternal stress on ethanol preference **(D)** or consumption **(E)** in JAX-sired male offspring. **(F)** Significant reduction in total fluid intake in JAX S-sired male offspring (*N* = 12/JAX groups and 7–12/PITT groups). *p < 0.05, **p < 0.01, ***p > 0.001.

In a two-bottle free choice ethanol drinking test, there was a significant interaction of paternal stress × sire source on both ethanol preference (*F*_(1,39)_ = 5.68, *p* < 0.05) and ethanol consumption (*F*_(1,39)_ = 5.36, *p* < 0.05). Therefore, the effects of paternal stress with both PITT-born and JAX-shipped sires were analyzed separately. For PITT-sired male offspring, there was a significant effect of ethanol concentration (*F*_(4,68)_ = 3.03, *p* < 0.01; Figure 9A) and no effect of paternal stress or paternal stress × ethanol concentration interaction on ethanol preference. There was a significant effect of ethanol concentration (*F*_(4,68)_ = 14.95, *p* < 0.001), paternal stress (*F*_(1,17)_ = 4.66, *p* < 0.05) and paternal stress × ethanol concentration (*F*_(4,68)_ = 3.22, *p* < 0.05; Figure 9B) on ethanol consumption. *Post hoc* analysis revealed reduced ethanol consumption at 12% (*p* < 0.05) and 15% (*p* < 0.001) ethanol concentrations in PITT S-sired males compared to Pitt C-sired males. There was a significant effect of ethanol concentration (*F*_(4,68)_ = 3.86, *p* < 0.01), but no effect of paternal stress or paternal stress × ethanol concentration on total fluid intake for PTT-sired offspring (Figure 9C). For male offspring of JAX-shipped sires, there was no effect of paternal stress or paternal stress × ethanol concentration on ethanol preference (Figure 9D). There was a significant effect of ethanol concentration (*F*_(4,88)_ = 33.87, *p* < 0.001), but not paternal stress or paternal stress × ethanol concentration on ethanol consumption (Figure 9E). For total fluid intake, there were significant effects of ethanol concentration (*F*_(4,88)_ = 3.42, *p* < 0.05), paternal stress (*F*_(1,22)_ = 9.20, *p* < 0.01) and paternal stress × ethanol concentration (*F*_(4,88)_ = 2.80, *p* < 0.05; Figure 9F). *Post hoc* analysis revealed a significant increase in total fluid intake at ethanol concentrations of 6, 9, 12 and 15% for S-sired vs. C-sired males (*p* < 0.05 for 6 and 9%, *p* < 0.01 for 12%, *p* < 0.001 for 15%).

### Paternal Chronic Stress Reduces Binge-Like Ethanol Consumption Selectively in PITT-Sired Offspring

**Figure 10:**
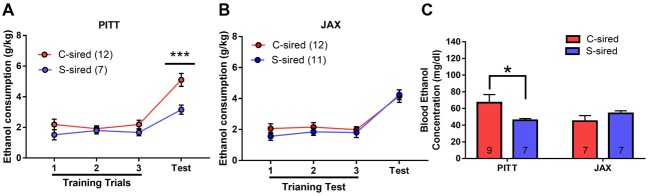
Sire-source dependent paternal stress reduces binge-like ethanol drinking. **(A)** Significant effect of paternal chronic variable stress on ethanol consumption in PITT-sired offspring. **(B)** No effect of JAX pre-stress sire conditions on ethanol consumption. **(C)** Significant effect of paternal chronic stress on blood ethanol concentrations following the 4-h drinking in the dark test trial for PITT, but not JAX S-sired vs. C-sired male offspring (*N* = 7–12/group for PITT-sired males and 12/group for JAX-sired males). *p < 0.05, ***p < 0.001.

In the drinking in the dark assay, there was a significant effect of paternal stress (*F*_(1,38)_ = 7.05, *p* < 0.05), paternal stress × sire source (*F*_(1,38)_ = 4.65, *p* < 0.05), and paternal stress × sire source × trial (*F*_(1,38)_ = 5.75, *p* < 0.05) on ethanol consumption (Figure 10). Again, the mice from PITT and JAX-shipped sires were analyzed separately. For male offspring from PITT sires, there was a significant effect of trial (*F*_(3,51)_ = 27.48, *p* < 0.001), paternal stress (*F*_(1,17)_ = 10.91, *p* < 0.01) and paternal stress × trial (*F*_(1,17)_ = 6.12, *p* < 0.05; Figure 10A). *Post hoc* analysis revealed significantly reduced ethanol consumption during the 4-h test in PITT S-sired vs. C-sired males (*p* < 0.001). For male offspring from JAX sires, there was a significant effect of trial (*F*_(3,63)_ = 42.40, *p* < 0.001), but no effect of paternal stress or paternal stress × trial on ethanol consumption (Figure 10B).

When we examined BECs immediately following the 4-h test trial, there was no effect of paternal stress or sire source, but there was a significant paternal stress × sire source interaction (*F*_(1,26)_ = 4.78, *p* < 0.05; Figure 10C). *Post hoc* analyses revealed reduced BECs for S-sired vs. C-sired male offspring from PITT sires (*p* < 0.05) and no difference in BECs for S-sired vs. C-sired male offspring from JAX sires.

## Discussion

In the present study, we found that paternal preconception chronic variable stress exposure attenuated ethanol drinking behavior selectively in male offspring. This intergenerational effect was specifically imparted by sires born within the in-house colony; there was no effect of paternal stress on offspring ethanol drinking when sires were born and shipped from the vendor during adulthood. Overall, the present results support the hypothesis that chronic preconception stress imparts unique ethanol drinking phenotypes to male offspring, although this intergenerational effect is dependent on the pre-stress environment of sires.

Paternal chronic variable stress significantly reduced ethanol drinking behavior in two distinct ethanol drinking paradigms, the two-bottle choice ethanol drinking test with continuous access (Figures 2, 9) and the limited access drinking in the dark test that models binge-like drinking (Figures 3, 10). The reduction in two-bottle choice ethanol drinking in stress-sired males, as well as the increase in adult body weight, were strikingly similar to the intergenerational effects of paternal chronic intermittent ethanol exposure (Finegersh and Homanics, 2014; Rompala et al., 2017). As in those studies, reduced ethanol drinking behavior was selective to male offspring and specific to ethanol, as water, saccharine, and quinine drinking were unaltered. This is consistent with previous studies with paternal ethanol exposure where only male offspring exhibited the reduced ethanol preference and consumption in the two-bottle choice test (Finegersh and Homanics, 2014; Rompala et al., 2017). While many other studies have found sex-specific intergenerational effects (Vassoler et al., 2013a,b)—either affecting just males or females—the mechanisms remain to be elucidated. It is worth noting that there were effects of paternal stress on total fluid intake in the two-bottle choice test for PITT-sired females (Figure 2F) as well as JAX-sired stress males (Figure 9F). Future studies are needed to further explore these phenotypes as differences in total fluid intake may be affected by relevant stress-related factors such as single-housing during the behavioral testing period (Scalera, 1992).

It is unclear exactly how chronic variable stress and chronic intermittent ethanol exposure impart similar intergenerational effects on ethanol drinking behavior. Each chronic variable stressor in the present study, as well as ethanol exposure, significantly increases CORT levels (Lee and Rivier, 2003; Willner, 2017). Moreover, both chronic stress and ethanol exposures reshape glucocorticoid receptor expression throughout the central nervous system (Vendruscolo et al., 2012; Guidotti et al., 2013; Willner, 2017). Fittingly, chronic variable stress and chronic intermittent ethanol exposures have similar effects on HPA responsivity to acute restraint stress in the next generation (Rodgers et al., 2013; Rompala et al., 2016), although the present study failed to reproduce the results from Rodgers et al. (2013; discussed below). As CORT facilitates ethanol drinking behavior in rodents (Fahlke et al., 1994, 1996; Fahlke and Eriksson, 2000), HPA axis hyporesponsivity may contribute to intergenerational ethanol drinking behavior in both paternal exposure paradigms. Thus, further study of these two unique sire exposures may be advantageous for identifying shared heritable epigenetic alterations in the offspring brain that drive reduced intergenerational ethanol drinking behavior.

The other major finding was the dependence of intergenerational ethanol drinking behaviors on the environment of the sire prior to chronic stress exposure during adulthood. Specifically, while all studies utilized B6 sires that were originally sourced from JAX, some sires were the first generation of an in-house colony whereas others were born at JAX and shipped 1 week prior to the onset of chronic variable stress. Remarkably, in-house (i.e., PITT) stressed-sires imparted reduced ethanol drinking behaviors to male offspring, while there were no intergenerational effects observed in offspring of JAX stressed-sires (Figures 9, 10). This finding suggests that PITT and JAX sires differentially responded to chronic variable stress. Supporting this notion, 1 week following chronic stress exposure, basal CORT levels were increased in JAX sires, but not in PITT sires. Relatedly, paternal preconception social defeat stress differentially affects offspring CORT and social behavior depending on whether sires were determined to be susceptible or resilient to social defeat (Dietz et al., 2011). Thus, differences in the sire environment such as shipping immediately prior to chronic stress may have shaped resilience or vulnerability to stress that, in turn, imparted disparate ethanol drinking behaviors to offspring.

There is an extensive literature directly examining the effects of animal shipping history. For instance, shipping stress increased blood pressure for up to 3 weeks after shipping in mice (Hoorn et al., 2011). Moreover, mice shipped at 6 weeks old vs. 12 weeks old showed reduced sexual behavior and CORT in adulthood (Laroche et al., 2009; Ismail et al., 2011). Finally, shipping during adolescence has been found to alter drug sensitivity and several metabolic and immunological measures (Bean-Knudsen and Wagner, 1987; Wiley and Evans, 2009). The present study expands on this work by demonstrating that cross-generational phenotypes are also potentially impacted by shipping stress. In addition to shipping stress, minor differences in the postnatal and adolescent environment may also shape adult physiology. For instance, HPA axis responsivity was found to vary between animal vendors (Turnbull and Rivier, 1999; Pecoraro et al., 2006; Olfe et al., 2010). Furthermore, vendor history plays a causal role in shaping the fecal microbiota of mice (Ericsson et al., 2015), and microbiota alterations influence stress-related behaviors (Foster and McVey Neufeld, 2013). Thus, future studies will need to directly examine PITT vs. JAX sires for various predisposing adaptations in stress physiology that may predict chronic variable stress vulnerability and intergenerational phenotypes.

Surprisingly, there was no effect of paternal chronic variable stress on male and female offspring HPA axis responsivity, in contrast to what has previously been reported (Rodgers et al., 2013). Due to mouse number limitations, HPA axis responsivity was examined in mice that 2 weeks prior underwent the drinking in the dark test. Thus, it is possible that the effects of paternal chronic variable stress on HPA axis responsivity were masked by the preceding behavioral experience. In addition, in the present study, B6 sires were bred with Strain 129 females to produce B6 × Strain 129 hybrid offspring. In Rodgers et al. (2013) both sires and breeder females were on a B6 × Strain 129 mixed background. Therefore, the effects of chronic variable stress may differ between B6 and B6 × 129 males given the differences in stress responsivity between these two strains (van Bogaert et al., 2006; Chan et al., 2017). Moreover, B6 and Strain 129 females exhibit different levels of maternal care (Champagne et al., 2007). Therefore, direct comparison between the present study and Rodgers et al. (2013) must be carefully considered.

Although the effects of paternal chronic stress mirror those of paternal chronic ethanol exposure on ethanol drinking behavior in the next generation (Finegersh and Homanics, 2014; Rompala et al., 2017), the phenomenon of attenuated intergenerational ethanol drinking behavior is surprising given the high heritability associated with alcohol use disorder (Verhulst et al., 2015). Nevertheless, similar “resilient”-like phenotypes have been reported following paternal cocaine exposure and paternal chronic stress where offspring exhibited reduced cocaine seeking behavior and stress responsivity, respectively (Rodgers et al., 2013; Vassoler et al., 2013b). Thus, it is possible that the paternal environment may have an unappreciated buffering role in the heritability of psychiatric disease and addiction. Conversely, no single model of stress or drug exposure can fully model complex human pathologies and, therefore, the heritable effects of paternal preconception environment may be sensitive to the exposure paradigm. For instance, one recent study found that paternal cocaine could both significantly increase or decrease cocaine taking in male offspring, depending on whether the paternal preconception exposure was voluntary or involuntary, respectively (Le et al., 2017). Overall, more studies are needed to fully appreciate how the paternal environment affects intergenerational ethanol drinking behavior.

Many studies have implicated germline epigenetic alterations in the intergenerational effects of paternal preconception environment. For instance, postnatal maternal separation and chronic variable stress alter several sperm microRNAs in rodents (Rodgers et al., 2013; Gapp et al., 2014) and alterations in sperm miRNA were recently reported in adult men exposed to early life stress (Dickson et al., 2018). Remarkably, in Rodgers et al. (2015) chronic variable stress with mice increased nine microRNAs in sperm that, when injected into normal fertilized embryos, recapitulated the intergenerational effects of paternal stress on HPA axis responsivity. Whether the same microRNAs are enriched in stressed PITT or JAX sires in the present study is unknown. Notably, chronic intermittent ethanol vapor exposure does not affect any of the nine stress-enriched miRNAs (Rompala et al., 2018). Thus, other small noncoding RNA types in sperm, such as tRNA-derived small noncoding RNAs (Chen et al., 2016; Sharma et al., 2016), or alternative epigenetic mechanisms such as DNA methylation and histone modifications are more likely to underlie intergenerational ethanol drinking behaviors.

In summary, the present study expands the rapidly growing number of effects associated with paternal preconception stress to include reduced ethanol drinking behavior. These results suggest the interwoven mechanisms of stress and ethanol extend across generations. By illuminating the significance of paternal preconception environment on ethanol drinking behavior, these findings have important implications for determining familial risk of addiction disorders with complex behavioral symptomology.

## Author Contributions

GR and GH designed the experiments, analyzed the data and wrote the manuscript. GR, AS and BK performed the experiments.

## Conflict of Interest Statement

The authors declare that the research was conducted in the absence of any commercial or financial relationships that could be construed as a potential conflict of interest. The reviewer PB and handling editor declared their shared affiliation at time of review.

## Supplementary Material

FIGURE S1 No effect of paternal stress on saccharin or quinine preference in a two bottle choice, unlimited access drinking assay. **(A)** No effect of paternal stress on saccharin or quinine drinking preference in male offspring (*N* = 8/group). **(B)** No effect of paternal stress on saccharin and quinine preference in female offspring (*N* = 8/group).

## References

[B1] Bean-Knudsen D. E. Wagner J. E. (1987). Effect of shipping stress on clincopathologic indicators in F344/N rats. Am. J. Vet. Res. 48, 306–308. 3826873

[B2] Becker H. C. Lopez M. F. Doremus-Fitzwater T. L. (2011). Effects of stress on alcohol drinking: a review of animal studies. Psychopharmacology 218, 131–156. 10.1007/s00213-011-2443-9 21850445 PMC3247761

[B3] Bowers M. E. Yehuda R. (2016). Intergenerational transmission of stress in humans. Neuropsychopharmacology 41, 232–244. 10.1038/npp.2015.247 26279078 PMC4677138

[B4] Champagne F. A. Curley J. P. Keverne E. B. Bateson P. P. (2007). Natural variations in postpartum maternal care in inbred and outbred mice. Physiol. Behav. 91, 325–334. 10.1016/j.physbeh.2007.03.014 17477940

[B5] Chan J. C. Houghton A. B. Bale T. L. (2017). Strained in planning your mouse background? Using the HPA stress axis as a biological readout for backcrossing strategies. Neuropsychopharmacology 42, 1749–1751. 10.1038/npp.2017.66 28361869 PMC5520787

[B6] Chan J. C. Nugent B. M. Bale T. L. (2018). Parental advisory: maternal and paternal stress can impact offspring neurodevelopment. Biol. Psychiatry 83, 886–894. 10.1016/j.biopsych.2017.10.005 29198470 PMC5899063

[B7] Chen Q. Yan M. Cao Z. Li X. Zhang Y. Shi J.. (2016). Sperm tsRNAs contribute to intergenerational inheritance of an acquired metabolic disorder. Science 351, 397–400. 10.1126/science.aad7977 26721680

[B8] Dickson D. A. Paulus J. K. Mensah V. Lem J. Saavedra-Rodriguez L. Gentry A.. (2018). Reduced levels of miRNAs 449 and 34 in sperm of mice and men exposed to early life stress. Transl. Psychiatry 8:101. 10.1038/s41398-018-0146-2 29795112 PMC5966454

[B9] Dietz D. M. Laplant Q. Watts E. L. Hodes G. E. Russo S. J. Feng J.. (2011). Paternal transmission of stress-induced pathologies. Biol. Psychiatry 70, 408–414. 10.1016/j.biopsych.2011.05.005 21679926 PMC3217197

[B10] Ericsson A. C. Davis J. W. Spollen W. Bivens N. Givan S. Hagan C. E.. (2015). Effects of vendor and genetic background on the composition of the fecal microbiota of inbred mice. PLoS One 10:e0116704. 10.1371/journal.pone.0116704 25675094 PMC4326421

[B12] Fahlke C. Engel J. A. Eriksson C. J. Hård E. Söderpalm B. (1994). Involvement of corticosterone in the modulation of ethanol consumption in the rat. Alcohol 11, 195–202. 10.1016/0741-8329(94)90031-0 8060519

[B11] Fahlke C. Eriksson C. J. (2000). Effect of adrenalectomy and exposure to corticosterone on alcohol intake in alcohol-preferring and alcohol-avoiding rat lines. Alcohol Alcohol. 35, 139–144. 10.1093/alcalc/35.2.139 10787388

[B13] Fahlke C. Hård E. Hansen S. (1996). Facilitation of ethanol consumption by intracerebroventricular infusions of corticosterone. Psychopharmacology 127, 133–139. 10.1007/bf02805986 8888379

[B15] Finegersh A. Homanics G. E. (2014). Paternal alcohol exposure reduces alcohol drinking and increases behavioral sensitivity to alcohol selectively in male offspring. PLoS One 9:e99078. 10.1371/journal.pone.0099078 24896617 PMC4045990

[B14] Finegersh A. Rompala G. R. Martin D. I. Homanics G. E. (2015). Drinking beyond a lifetime: new and emerging insights into paternal alcohol exposure on subsequent generations. Alcohol 49, 461–470. 10.1016/j.alcohol.2015.02.008 25887183 PMC4469624

[B16] Foster J. A. McVey Neufeld K. A. (2013). Gut-brain axis: how the microbiome influences anxiety and depression. Trends Neurosci. 36, 305–312. 10.1016/j.tins.2013.01.005 23384445

[B17] Franklin T. B. Russig H. Weiss I. C. Gräff J. Linder N. Michalon A.. (2010). Epigenetic transmission of the impact of early stress across generations. Biol. Psychiatry 68, 408–415. 10.1016/j.biopsych.2010.05.036 20673872

[B18] Gapp K. Bohacek J. Grossmann J. Brunner A. M. Manuella F. Nanni P.. (2016). Potential of environmental enrichment to prevent transgenerational effects of paternal trauma. Neuropsychopharmacology 41, 2749–2758. 10.1038/npp.2016.87 27277118 PMC5026744

[B19] Gapp K. Jawaid A. Sarkies P. Bohacek J. Pelczar P. Prados J.. (2014). Implication of sperm RNAs in transgenerational inheritance of the effects of early trauma in mice. Nat. Neurosci. 17, 667–669. 10.1038/nn.3695 24728267 PMC4333222

[B20] Guidotti G. Calabrese F. Anacker C. Racagni G. Pariante C. M. Riva M. A. (2013). Glucocorticoid receptor and FKBP5 expression is altered following exposure to chronic stress: modulation by antidepressant treatment. Neuropsychopharmacology 38, 616–627. 10.1038/npp.2012.225 23169346 PMC3572458

[B21] Haberstick B. C. Young S. E. Zeiger J. S. Lessem J. M. Hewitt J. K. Hopfer C. J. (2014). Prevalence and correlates of alcohol and cannabis use disorders in the United States: results from the national longitudinal study of adolescent health. Drug Alcohol Depend. 136, 158–161. 10.1016/j.drugalcdep.2013.11.022 24440049 PMC3963405

[B22] Hoorn E. J. McCormick J. A. Ellison D. H. (2011). High tail-cuff blood pressure in mice 1 week after shipping: the need for longer acclimation. Am. J. Hypertens. 24, 534–536. 10.1038/ajh.2011.7 21293389 PMC3740725

[B23] Ismail N. Garas P. Blaustein J. D. (2011). Long-term effects of pubertal stressors on female sexual receptivity and estrogen receptor-α expression in CD-1 female mice. Horm. Behav. 59, 565–571. 10.1016/j.yhbeh.2011.02.010 21376052 PMC3085923

[B24] Jones R. C. (1999). To store or mature spermatozoa? The primary role of the epididymis. Int. J. Androl. 22, 57–67. 10.1046/j.1365-2605.1999.00151.x 10194636

[B25] Laroche J. Gasbarro L. Herman J. P. Blaustein J. D. (2009). Reduced behavioral response to gonadal hormones in mice shipped during the peripubertal/adolescent period. Endocrinology 150, 2351–2358. 10.1210/en.2008-1595 19131570 PMC2671909

[B26] Le Q. M. Yan B. Yu X. C. Li Y. Q. Song H. K. Zhu H. W.. (2017). Drug-seeking motivation level in male rats determines offspring susceptibility or resistance to cocaine-seeking behaviour. Nat. Commun. 8:15527. 10.1038/ncomms15527 28556835 PMC5459992

[B27] Lee S. Rivier C. (2003). Long-term influence of an initial exposure to alcohol on the rat hypothalamic-pituitary axis. Alcohol. Clin. Exp. Res. 27, 1463–1470. 10.1097/01.alc.0000086065.06203.dd 14506408

[B28] Olfe J. Domanska G. Schuett C. Kiank C. (2010). Different stress-related phenotypes of BALB/c mice from in-house or vendor: alterations of the sympathetic and HPA axis responsiveness. BMC Physiol. 10:2. 10.1186/1472-6793-10-2 20214799 PMC2845127

[B29] Pecoraro N. Ginsberg A. B. Warne J. P. Gomez F. la Fleur S. E. Dallman M. F. (2006). Diverse basal and stress-related phenotypes of Sprague Dawley rats from three vendors. Physiol. Behav. 89, 598–610. 10.1016/j.physbeh.2006.07.019 16935312

[B30] Pisu M. G. Garau A. Olla P. Biggio F. Utzeri C. Dore R.. (2013). Altered stress responsiveness and hypothalamic-pituitary-adrenal axis function in male rat offspring of socially isolated parents. J. Neurochem. 126, 493–502. 10.1111/jnc.12273 23600845

[B31] Rehm J. Mathers C. Popova S. Thavorncharoensap M. Teerawattananon Y. Patra J. (2009). Alcohol and Global Health 1 Global burden of disease and injury and economic cost attributable to alcohol use and alcohol-use disorders. Lancet 373, 2223–2233. 10.1016/s0140-6736(09)60746-7 19560604

[B32] Rivier C. (2014). Role of hypothalamic corticotropin-releasing factor in mediating alcohol-induced activation of the rat hypothalamic-pituitary-adrenal axis. Front. Neuroendocrinol. 35, 221–233. 10.1016/j.yfrne.2013.10.005 24211830

[B33] Rodgers A. B. Morgan C. P. Bronson S. L. Revello S. Bale T. L. (2013). Paternal stress exposure alters sperm microRNA content and reprograms offspring HPA stress axis regulation. J. Neurosci. 33, 9003–9012. 10.1523/JNEUROSCI.0914-13.2013 23699511 PMC3712504

[B34] Rodgers A. B. Morgan C. P. Leu N. A. Bale T. L. (2015). Transgenerational epigenetic programming via sperm microRNA recapitulates effects of paternal stress. Proc. Natl. Acad. Sci. U S A 112, 13699–13704. 10.1073/pnas.1508347112 26483456 PMC4640733

[B35] Rompala G. R. (2018). Role of Paternal Preconception Environment in Ethanol- and Stress-Related Phenotypes [Dissertation]. Pittsburgh, PA: University of Pittsburgh.

[B36] Rompala G. R. Finegersh A. Homanics G. E. (2016). Paternal preconception ethanol exposure blunts hypothalamic-pituitary-adrenal axis responsivity and stress-induced excessive fluid intake in male mice. Alcohol 53, 19–25. 10.1016/j.alcohol.2016.03.006 27286933 PMC4904231

[B37] Rompala G. R. Finegersh A. Slater M. Homanics G. E. (2017). Paternal preconception alcohol exposure imparts intergenerational alcohol-related behaviors to male offspring on a pure C57BL/6J background. Alcohol 60, 169–177. 10.1016/j.alcohol.2016.11.001 27876231 PMC5419883

[B38] Rompala G. R. Mounier A. Wolfe C. M. Lin Q. Lefterov I. Homanics G. E. (2018). Heavy chronic intermittent ethanol exposure alters small noncoding RNAs in mouse sperm and epididymosomes. Front. Genet. 9:32. 10.3389/fgene.2018.00032 29472946 PMC5809758

[B39] Scalera G. (1992). Taste preferences, body-weight gain, food and fluid intake in singly or group-housed rats. Physiol. Behav. 52, 935–943. 10.1016/0031-9384(92)90374-b 1484850

[B40] Sharma U. Conine C. C. Shea J. M. Boskovic A. Derr A. G. Bing X. Y.. (2016). Biogenesis and function of tRNA fragments during sperm maturation and fertilization in mammals. Science 351, 391–396. 10.1126/science.aad6780 26721685 PMC4888079

[B41] Short A. K. Fennell K. A. Perreau V. M. Fox A. O’Bryan M. K. Kim J. H.. (2016). Elevated paternal glucocorticoid exposure alters the small noncoding RNA profile in sperm and modifies anxiety and depressive phenotypes in the offspring. Transl. Psychiatry 6:e837. 10.1038/tp.2016.109 27300263 PMC4931607

[B42] Tambour S. Brown L. L. Crabbe J. C. (2008). Gender and age at drinking onset affect voluntary alcohol consumption but neither the alcohol deprivation effect nor the response to stress in mice. Alcohol. Clin. Exp. Res. 32, 2100–2106. 10.1111/j.1530-0277.2008.00798.x 18828803

[B43] Thiele T. E. Crabbe J. C. Boehm S. L. II. (2014). “Drinking in the Dark” (DID): a simple mouse model of binge-like alcohol intake. Curr. Protoc. Neurosci. 68, 9.49.1–9.49.12. 10.1002/0471142301.ns0949s68 24984686 PMC4142649

[B44] Turnbull A. V. Rivier C. L. (1999). Sprague-Dawley rats obtained from different vendors exhibit distinct adrenocorticotropin responses to inflammatory stimuli. Neuroendocrinology 70, 186–195. 10.1159/000054475 10516481

[B45] van Bogaert M. J. Groenink L. Oosting R. S. Westphal K. G. van der Gugten J. Olivier B. (2006). Mouse strain differences in autonomic responses to stress. Genes Brain Behav. 5, 139–149. 10.1111/j.1601-183x.2005.00143.x 16507005

[B46] Vassoler F. M. Johnson N. L. Byrnes E. M. (2013a). Female adolescent exposure to cannabinoids causes transgenerational effects on morphine sensitization in female offspring in the absence of *in utero* exposure. J. Psychopharmacol. 27, 1015–1022. 10.1177/0269881113503504 24048098 PMC4262825

[B47] Vassoler F. M. White S. L. Schmidt H. D. Sadri-Vakili G. Pierce R. C. (2013b). Epigenetic inheritance of a cocaine-resistance phenotype. Nat. Neurosci. 16, 42–47. 10.1038/nn.3280 23242310 PMC3531046

[B48] Vendruscolo L. F. Barbier E. Schlosburg J. E. Misra K. K. Whitfield T. W. Jr. Logrip M. L.. (2012). Corticosteroid-dependent plasticity mediates compulsive alcohol drinking in rats. J. Neurosci. 32, 7563–7571. 10.1523/JNEUROSCI.0069-12.2012 22649234 PMC3375621

[B49] Verhulst B. Neale M. C. Kendler K. S. (2015). The heritability of alcohol use disorders: a meta-analysis of twin and adoption studies. Psychol. Med. 45, 1061–1072. 10.1017/s0033291714002165 25171596 PMC4345133

[B50] Wiley J. L. Evans R. L. (2009). To breed or not to breed? Empirical evaluation of drug effects in adolescent rats. Int. J. Dev. Neurosci. 27, 9–20. 10.1016/j.ijdevneu.2008.11.002 19041390 PMC2637202

[B51] Willner P. (2017). The chronic mild stress (CMS) model of depression: history, evaluation and usage. Neurobiol. Stress 6, 78–93. 10.1016/j.ynstr.2016.08.002 28229111 PMC5314424

