# Supplementary figures and images for: Paternal Preconception Chronic Variable Stress Confers Attenuated Ethanol Drinking Behavior Selectively to Male Offspring in a Pre-Stress Environment Dependent Manner
Source: Front Behav Neurosci. 2018 Nov 2;12:257. doi: 10.3389/fnbeh.2018.00257 (PMC6225737; doi:10.3389/fnbeh.2018.00257)

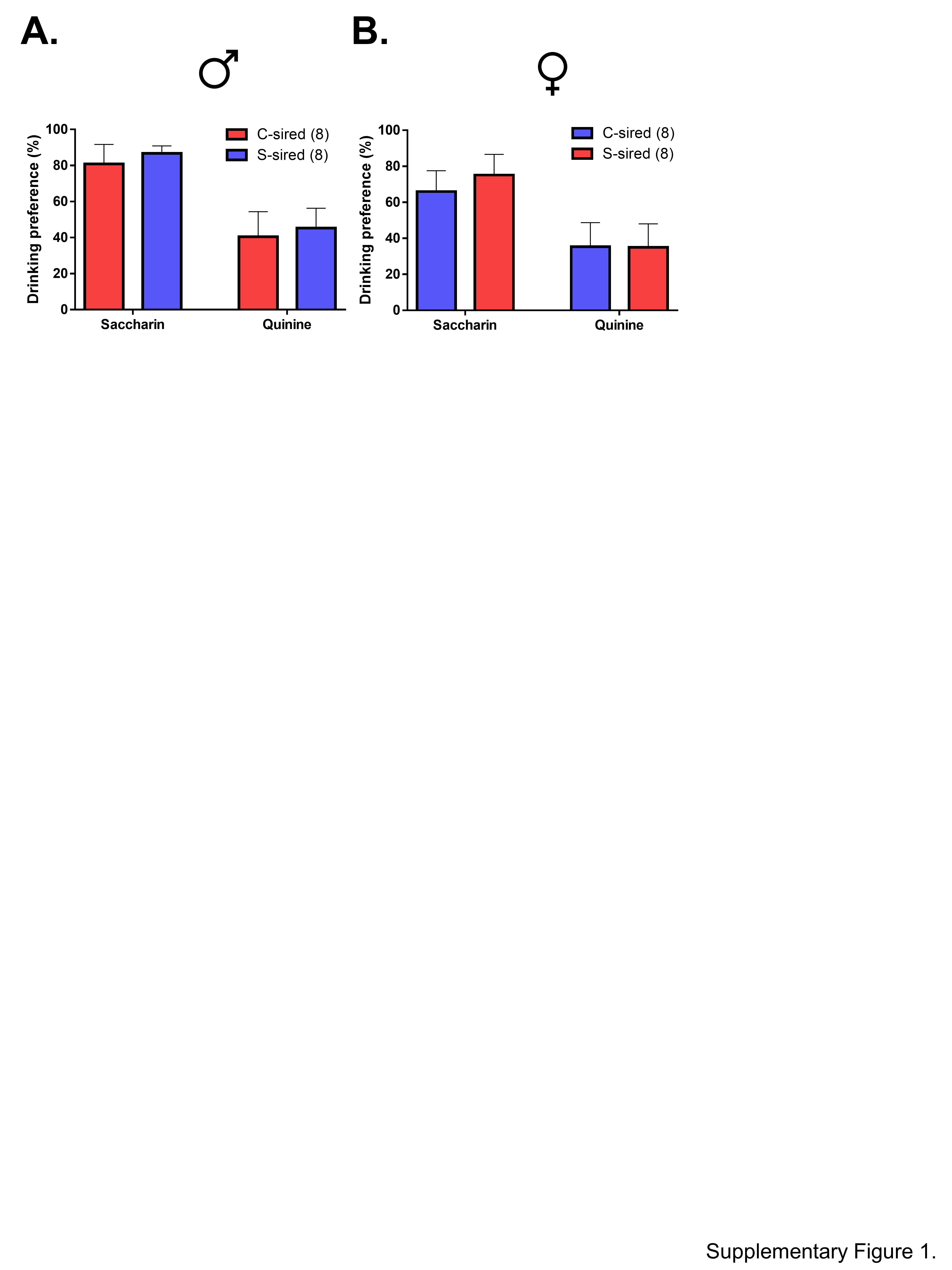

Supplement: FIGURE S1 — No effect of paternal stress on saccharin or quinine preference in a two bottle choice, unlimited access drinking assay. (A) No effect of paternal stress on saccharin or quinine drinking preference in male offspring (N = 8/group). (B) No effect of paternal stress on saccharin and quinine preference in female offspring (N = 8/group). [file Image_1.TIF]
