# Supplementary material for: Paternal Preconception Chronic Variable Stress Confers Attenuated Ethanol Drinking Behavior Selectively to Male Offspring in a Pre-Stress Environment Dependent Manner
Source: Front Behav Neurosci. 2018 Nov 2;12:257. doi: 10.3389/fnbeh.2018.00257 (PMC6225737; doi:10.3389/fnbeh.2018.00257)
Supplement: Supplementary file 2 [file Table_1.DOCX]

|  | Cohort 1 (PITT) | | Cohort 2 (JAX) | | Cohort 3 (PITT/JAX) | | | |
| --- | --- | --- | --- | --- | --- | --- | --- | --- |
| Treatment | Control | Stress | Control | Stress | PITT-Control | PITT-Stress | JAX-Control | JAX-Stress |
| Male Breeders | 8 | 8 | 16 | 16 | 10 | 10 | 8 | 8 |
| Litters | 6 | 7 | 14 | 13 | 14 | 9 | 9 | 8 |
| Sires with >1 Litter | 6 | 7 | 11 | 10 | 10 | 7 | 8 | 9 |
| Weaned Male Offspring | 17 | 20 | 39 | 50 | 19 | 7 | 19 | 14 |
| Weaned Female Offspring | 19 | 19 | 29 | 51 | only F1 males were examined in cohort 3 | | | |
| Paternal Stress Onset | January 2015 | | February 2016 | | January 2017 | | | |
| Notes | While in the same animal facility and floor, cohort 3 animals were housed in a different room from that of cohorts 1 and 2. | | | | | | | |

Supplementary Table 1: Experimental Cohort Summary. PITT= University of Pittsburgh. JAX= The Jackson Laboratory.
